# Integrated Cell-Based Transcriptomic and Pathway Profiling Enables Scalable Functional Screening of Bioactive Microalgal Extracts

**DOI:** 10.3390/ph19091440

**Published:** 2026-09-11

**Authors:** Boris Sorokin, Anton A. Buzdin, Daniil Luppov, Maksim Sorokin, Evgeniy Gusev, Roman Kholodenko, Elena Guglya, Zorigto Namsaraev, Ilia Yampolsky, Denis Kuzmin

**Affiliations:** 1 Moscow Center for Advanced Studies, 123592 Moscow, Russia; luppov.daniil17@gmail.com (D.L.); denisk@list.ru (D.K.); 2 Research Center for Genetics and Life Sciences, Sirius University of Science and Technology, 354340 Sirius, Russia; bu3din@mail.ru; 3 Burnasyan Federal Medical Biophysical Center, Federal Medical Biological Agency of Russia, 123098 Moscow, Russia; 4 Oncobox LLC, 141701 Dolgoprudny, Russia; sorokin.maks@gmail.com; 5 Severtsov Institute of Ecology and Evolution, Russian Academy of Sciences, Leninsky Prospect 33, 119071 Moscow, Russia; algogus@yandex.ru; 6 Shemyakin-Ovchinnikov Institute of Bioorganic Chemistry, Russian Academy of Sciences, 117997 Moscow, Russia; khol@mail.ru (R.K.); eguglya@gmail.com (E.G.); ivyamp@gmail.com (I.Y.); 7 Kurchatov Centre for Genome Research, National Research Centre “Kurchatov Institute”, 123182 Moscow, Russia

**Keywords:** microalgae, biotechnology, bioinformatics, molecular pathway analysis, systems biology, bioeconomy

## Abstract

**Background:** Microalgae and cyanobacteria represent a largely unexplored source of bioactive compounds, but systematic screening of extensive strain collections is limited by the cost and complexity of conventional chemical and pharmacological characterization. Here, we evaluated a scalable functional screening strategy integrating human cell-based gene expression profiling, quantitative molecular pathway analysis, and direct cytotoxicity testing to prioritize bioactive microalgal and cyanobacterial extracts for subsequent investigation. **Results:** Forty-eight extracts were screened using HL-60 human leukemia cells, and eight demonstrated detectable cytotoxic activity. Pathway-based prediction showed 92% agreement with direct cytotoxicity measurements. To assess whether pathway profiling could provide information on mechanisms of action, we performed an in-depth analysis of the most active *Nostoc* sp. SBV-48 extract and the control antimicrobial peptide Polyphemusin III. The inferred pathway responses were consistent with their previously reported mechanisms of cytotoxicity. Fractionation and chemical characterization of the *Nostoc* sp. SBV-48 extract identified Cryptophycin-1 as its major cytotoxic component, illustrating the intended sequential workflow from primary screening to characterization of prioritized hits. In addition, previously unreported cytotoxic activity against HL-60 cells was detected in three Desmidiales strains belonging to the genera *Closterium*, *Cosmarium*, and *Spondylosium*, which showed partially convergent pathway-level responses. **Conclusions:** Overall, the proposed approach provides a cost-effective first-stage strategy for functional screening and prioritization of complex microalgal extracts, allowing resource-intensive chemical characterization and broader pharmacological validation to be focused on the most promising candidates.

## 1. Introduction

Microalgae possess considerable biotechnological potential, as they are among the fastest-growing photosynthetic microorganisms on Earth [1,2]. Their cultivation requires relatively small amounts of water and can be carried out in areas unsuitable for agriculture. Furthermore, the growth of microalgae biomass is accompanied by the absorption of carbon dioxide and the release of oxygen [3]. Microalgae have the capacity to synthesize a diverse array of compounds, including proteins, carbohydrates, lipids, vitamins, and pigments. To date, researchers have successfully isolated more than 1000 biologically active substances from microalgae. These include compounds with antioxidant, antibacterial, antiviral, antitumor, regenerative, hypotensive, neuroprotective, and immunostimulatory properties [4]. The demand for bioproducts derived from microalgae is significant across multiple sectors, including agriculture, the chemical industry, energy, pharmacology, and medicine, and become especially relevant in the light of circular bioeconomy [5,6,7]. According to various expert estimates, the number of species of microalgae and cyanobacteria ranges from 200 to 800 thousand [8]. Presently, the Algaebase database (Listing the World’s Algae. 2024. URL: https://www.algaebase.org/; accessed on 1 May 2026) enumerates roughly 176 thousand species and intraspecies names. Microalgae and cyanobacteria are capable of rapid growth and can inhabit a wide range of ecological niches, which contributes to their exceptional physiological and biochemical diversity [9]. However, only a negligible portion of this diversity is utilized in modern biotechnology.

Studying microalgae is usually laborious and time-consuming. It requires preparing inoculum and setting up experiments with several variable parameters. Several weeks may be needed to conduct the experiments and accumulate the target product. Only then can the biomass undergo biochemical analysis, typically involving a limited number of predefined compounds. Each class of compounds requires its own specific methodology and instrumentation. Most often, known compounds with certain properties are analyzed [10,11]. Exploratory research aimed at finding new compounds with the desired properties requires more time and financial investment. Thus, developing approaches to screen collections of organisms and optimize the study of biochemical properties is a priority area of biotechnological research [12,13].

In this regard, modern Omics approaches seem promising because they can study molecular effects of unknown compounds in high-throughput manner. This allows researchers to select organisms with desired effects from strains and isolate and identify useful components [14,15,16,17,18]. Importantly, this approach is not limited to compounds with known functional or biochemical properties, thus increasing the likelihood of discovering new, more potent compounds.

A critical factor in optimizing and accelerating the screening of microalgae and other microorganisms is the analysis of a substantial number of strains within a constrained timeframe. The concurrent analysis of extensive panels of microorganisms not only accelerates the research process but also facilitates the collection of consistent data that are readily comparable [19]. The duration of the experiment is also a critical factor, as its prolongation has been shown to increase the likelihood of accumulating factors unrelated to the manipulated parameters but connected to the side effects of long-term cultivation. These effects include contamination in non-axenic cultures, the influence of secondary metabolite accumulation, and the decomposition of dead organisms. [20].

One of the most important issues in algae biotechnology is searching for valuable, productive strains that are capable of withstanding intensive cultivation and contamination [12,13,20]. However, most studies are limited to a narrow range of well-known model strains, so the majority of microalgal diversity remains unexplored. Concurrently, organisms belonging to novel and unstudied groups of microalgae frequently yield promising results and are regarded as biotechnologically valuable. For instance, a study of fucoxanthin in the members of the Order Synurales (class Chrysophyceae), specifically the genus *Mallomonas*, revealed the most effective fucoxanthin producer [21]. Thus, including new and unstudied groups of microalgae in biotechnological research may lead to the discovery of new valuable strains and active compounds.

A remaining challenge is the lack of integrated approaches that can rapidly screen complex microalgal extracts while simultaneously providing information on their potential molecular mechanisms of action. In this study, we addressed this gap by combining human cell-based functional testing with transcriptomic profiling and quantitative molecular pathway analysis. Previous applications of the Oncobox platform were primarily focused on cancer molecular profiling and personalized selection of antitumor therapies based on RNA or protein expression data [22,23]. Here, we extend this approach to the functional screening of complex microalgal and cyanobacterial extracts. The principal innovation is, therefore, not pathway analysis alone, but its integration with direct cytotoxicity testing to simultaneously prioritize biologically active extracts and obtain pathway-level information on their mechanisms of action.

## 2. Results

### 2.1. Rationale of the Study

The aim of the present study was to develop a bioinformatics platform for investigating the antitumor activity of microalgae extracts based on Oncobox technology. The platform utilizes an integrated model of human cell signalome to analyze the activation profiles of intracellular signaling pathways in cells treated with extracts. The obtained intracellular pathway activation levels (PALs) reflect the estimated activities of molecular pathways, where positive and negative values signify up- or downregulation of a pathway, and the absolute value of PAL reflects the extent of a pathway differential regulation [24]. The data can be used for the following purposes:

- Identifying the cytotoxic effect of the extract by analyzing the number of signaling pathways associated with cell death that are upregulated in test cells under the influence of the extract;
- Evaluating other biological activities of the extract and determining the mechanism of such activities by ranking the most strongly affected molecular pathways and analyzing the expression levels of individual genes within the pathway.

The experimental panel included 48 extracts of microalgae and cyanobacteria from nine distinct classes (Table 1, Supplementary Table S1), along with a control compound: the natural antimicrobial/antitumor peptide Polyphemusin III. The test extracts were incubated with HL-60 tumor cells in microplates. After incubation, we profiled gene expression by RNA microarrays in the affected and control cells. The differentially expressed genes were identified, and in parallel, molecular pathway activation patterns were quantitatively reconstructed using Oncobox method [25].

In order to validate Oncobox’s ability to detect cytotoxic activity based on the number of activated signaling pathways associated with cell death, direct measurement of cytotoxicity was performed on the same cell line using the MTT assay. To validate Oncobox’s ability to analyze the mechanisms of action of active compounds in extracts, two samples were selected for testing: the most cytotoxic *Nostoc* sp. SBV48 extract (from which the active metabolite Cryptophycin-1 was isolated) and the control compound Polyphemusin III. The results of the Oncobox analysis of the most altered signaling pathways were compared with the literature data on the mechanisms of action of cryptophycines and polyphemusines.

**Figure 1:**
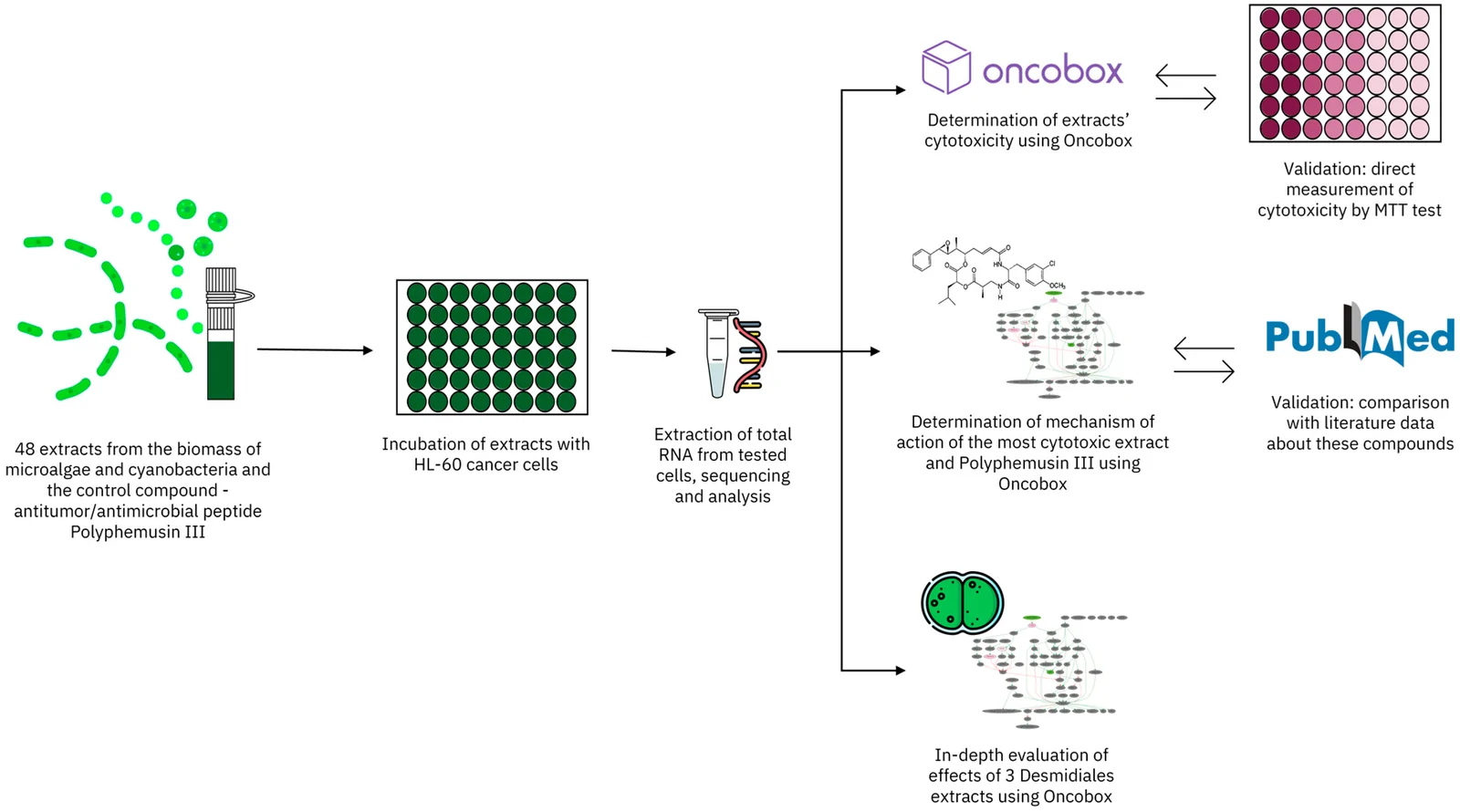
Design of the study.

Additionally, the study revealed the antitumor activity of three Desmidiales strains: *Closterium* sp. SBV86, *Cosmarium* sp. SBV90, and *Spondylosium planum* SBV92. The antitumor activity of microalgae from that order has not been previously described in the scientific literature. We analyzed detailed data on signaling pathway activation to investigate the mechanisms of this activity. The overall study design is shown on Figure 1.

### 2.2. Evaluation of In Vitro Cytotoxic Activities of Tested Extracts

Direct evaluation of cytotoxicity of the extracts was carried out by MTT assay on the same HL-60 cell line. β-hairpin antimicrobial peptide Polyphemusin III, isolated from the hemocytes of the horseshoe crab *Limulus polyphemus*, was used as a control natural cytotoxic agent. Its antitumor activity is connected to the disruption of the integrity of the cancer cell membrane and was extensively studied in previous work [26].

We found that eight extracts exhibited detectable cytotoxic activity (IC_50_ < 1 mg/mL). It was outstandingly high for the *Nostoc* sp. SBV48 extract, whose half-inhibiting cell growth concentration (IC_50_) was four orders of magnitude smaller than for Polyfemusin III (Table 2). For the remaining seven species, their IC_50_ was instead approximately 50–330 times greater than for Polyfemusin III (Table 2).

Out of the 48 samples tested, the Oncobox method identified 8 extracts that caused activation of signaling pathways associated with cell death in affected HL-60 cells (Table 2). Treatment of tumor cells with the control compound Polyphemusin III (IC_50_ of 2.5 ± 0.1 μM) also resulted in the activation of 3 such signaling pathways, which correlates well with previously published data [26].

The remaining 40 extracts showed no detectable cytotoxicity in either the MTT test or the molecular pathway activation assay. Only 4 of the 48 extracts showed discrepancies between the pathway-predicted and direct measurements of cytotoxicity. Overall, these results demonstrate a high accuracy (92%) with a 0% false-positives rate for pathway-based predictions in identifying in vitro antitumor activity in microalgal and cyanobacterial extracts.

### 2.3. Detailed Analysis of Molecular Pathways Affected by Extracts

To evaluate an ability to determine the mechanism of cytotoxic activity based solely on Oncobox transcriptomic data, we conducted detailed quantitative and qualitative analysis of signal pathway activation for the most cytotoxic extract from *Nostoc* sp. SBV48 (for which an active metabolite was identified and purified) and the control compound Polyphemusin III. The results obtained were then compared with the available literature data on the relevant mechanism of cytotoxic action of these compounds.

#### 2.3.1. Identification of an Active Compound in *Nostoc* sp. SBV48 Extract

Analysis of the NMR spectra revealed that the active component of *Nostoc* sp. SBV48 extract contained two aromatic rings—benzoic and phenolic, with the latter substituted in ortho- and para-sites. Additionally, the molecule contained two peptide bonds, two carboxyl groups, four CH-O groups, five methyl groups, four CH_2_-moieties, and a double bond in Z-configuration (^3^J_HH_ = 15 Hz). The complete structure was elucidated by analyzing a set of 2D homo- and heteronuclear experiments and is shown in the Results section. The structure is characterized by a molecular weight of 655.27 ([MH+]), which corresponds to the experimental value (*m*/*z* = 655.6). The full chemical shift assignment is provided in Supplementary Table S2. Supplementary Figure S1 provides the 1D ^13^C NMR spectrum of the compound with the signal assignment.

**Figure 2:**
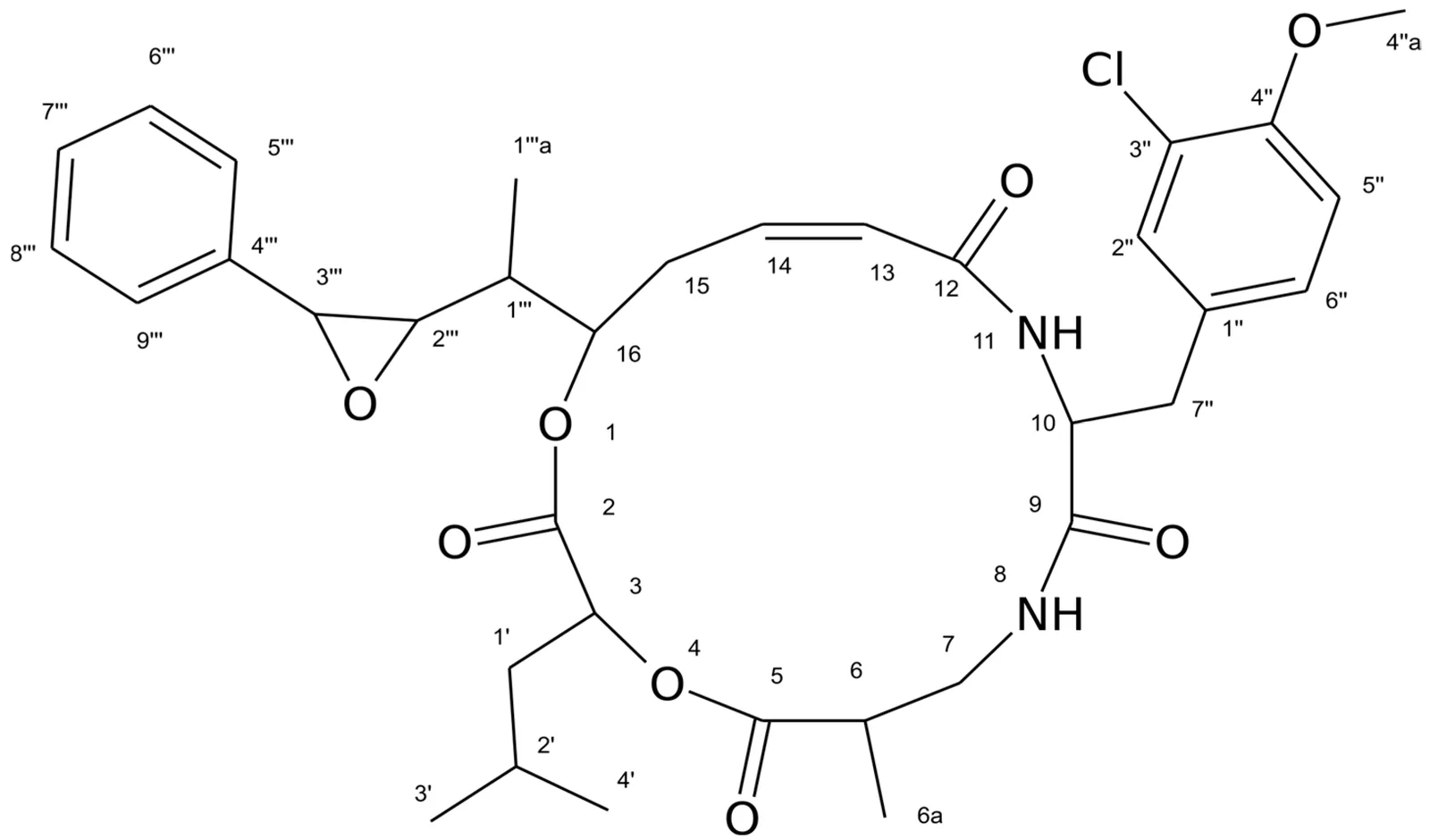
Structure of the active component of *Nostoc* sp. SBV48 extract (Cryptophycin-1) with the indication of atom numbering.

NMR structure identification revealed that the active cytotoxic compound of the *Nostoc* sp. SBV48 biomass extract was the cyanobacterial peptide toxin Cryptophycin-1 with only 0.01% mass concentration in the extract under analysis (Figure 2).

Cryptophycins are the major members of the class of macrocyclic depsipeptides. Cryptophycin was first isolated back in 1990 from blue-green algae (cyanobacteria) of the genus *Nostoc* (GSV 224) [27]. It exhibited cytotoxic effects on fungi and yeasts of the *Cryptococcus* genus, which gave its name to this class of compounds. In addition, most cryptocphycins have been shown to exhibit antitumor activity [28]. The proposed mechanism for the cytotoxic effect of cryptophycins is the depolarization of microtubules causing cell cycle arrest in the G1/M phase. To date, several different cryptophycins have been discovered, and their synthetic analogs have been obtained, some of which were examined in phase I−II clinical trials as the antitumor agents [29,30].

#### 2.3.2. Mechanisms of Cytotoxic Activity of Cryptophycin-1 from *Nostoc* sp. SBV48 Extract

Using the pathway interrogation approach, as many as 25 molecular signaling pathways associated with cell death were statistically significantly upregulated in the affected cells. Furthermore, among all the pathways, the two most strongly affected pathways were as follows: the Mitochondrial apoptosis pathway and the Integrin-linked kinase signaling (actin polymerization and cytoskeletal reorganization) pathway.

**Figure 3:**
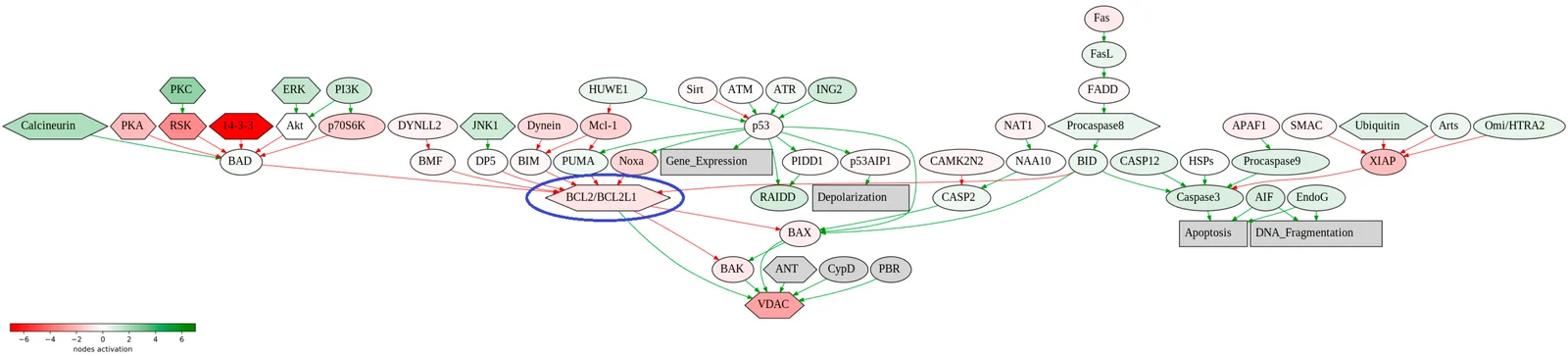
Mitochondrial apoptosis pathway activation diagram in response to the *Nostoc* sp. SBV48 extract. Green and red arrows correspond to node activation and inhibition, respectively. The color intensity of each node in the graph corresponds to the logarithm of the experimental-to-control ratio of gene expression.

In the previous studies, Cryptophycin-1 could inhibit anti-apoptotic function of Bcl-2 protein through its hyperphosphorylation [31,32,33]. In this study, in the tested samples treated with *Nostoc* sp. SBV48 extract, a strong activation of the Mitochondrial apoptotic pathway (PAL = 17.3) was observed, which was associated with the transcriptional inhibition of *BCL2* gene (Figure 3).

**Figure 4:**
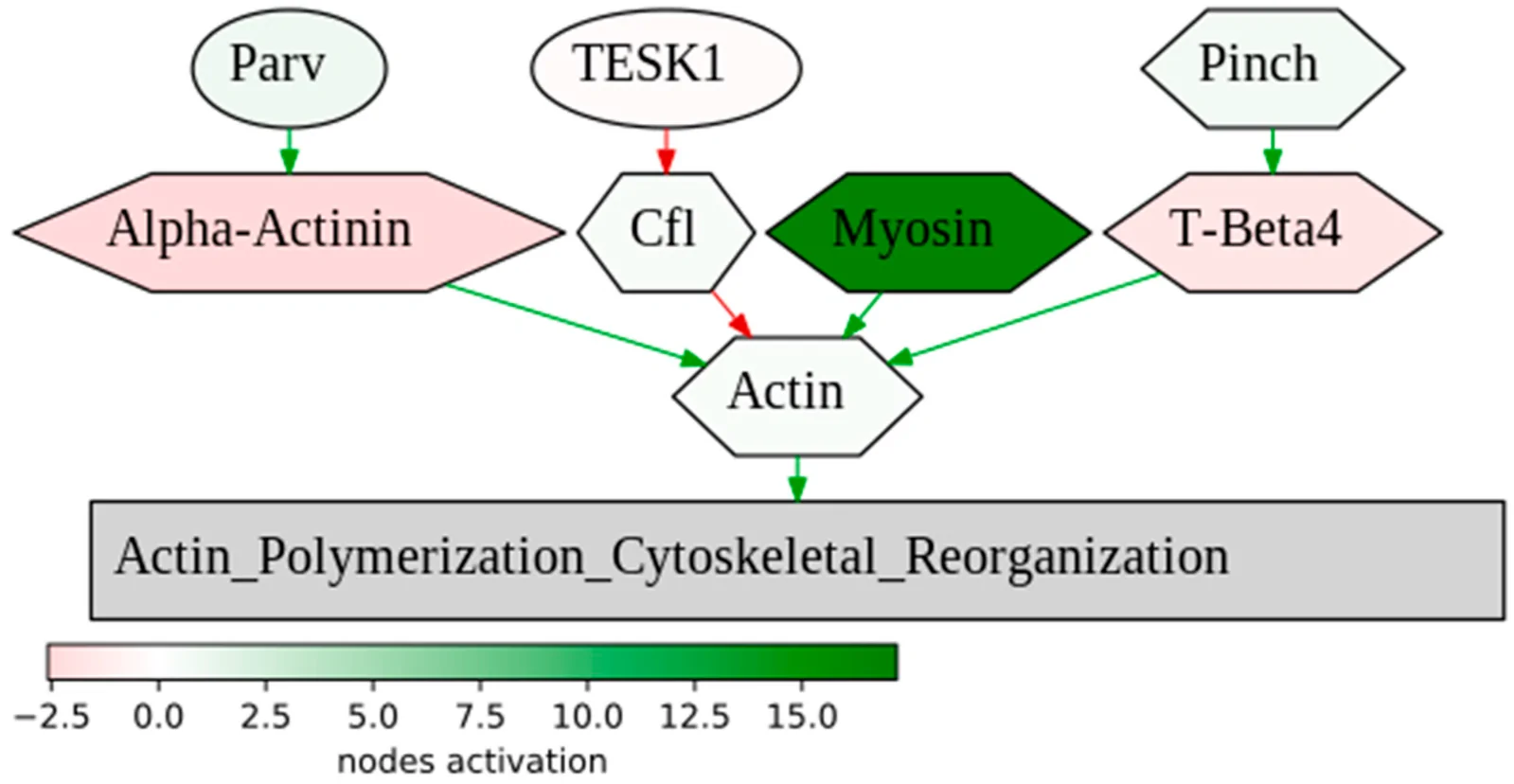
Integrin-linked kinase signaling (actin polymerization and cytoskeletal reorganization) pathway activation diagram in response to the *Nostoc* sp. SBV48 extract. Green and red arrows correspond to node activation and inhibition, respectively. The color intensity of each node in the graph corresponds to the logarithm of the experimental-to-control ratio of gene expression.

The observed activation of Rearrangement of the cytoskeleton pathway (PAL value of 18) could be a consequence of microtubule homeostasis disruption induced by the addition of Cryptophycin-1 [34], as shown in Figure 4.

#### 2.3.3. Mechanisms of Cytotoxic Activity of Polyphemusin III

**Figure 5:**
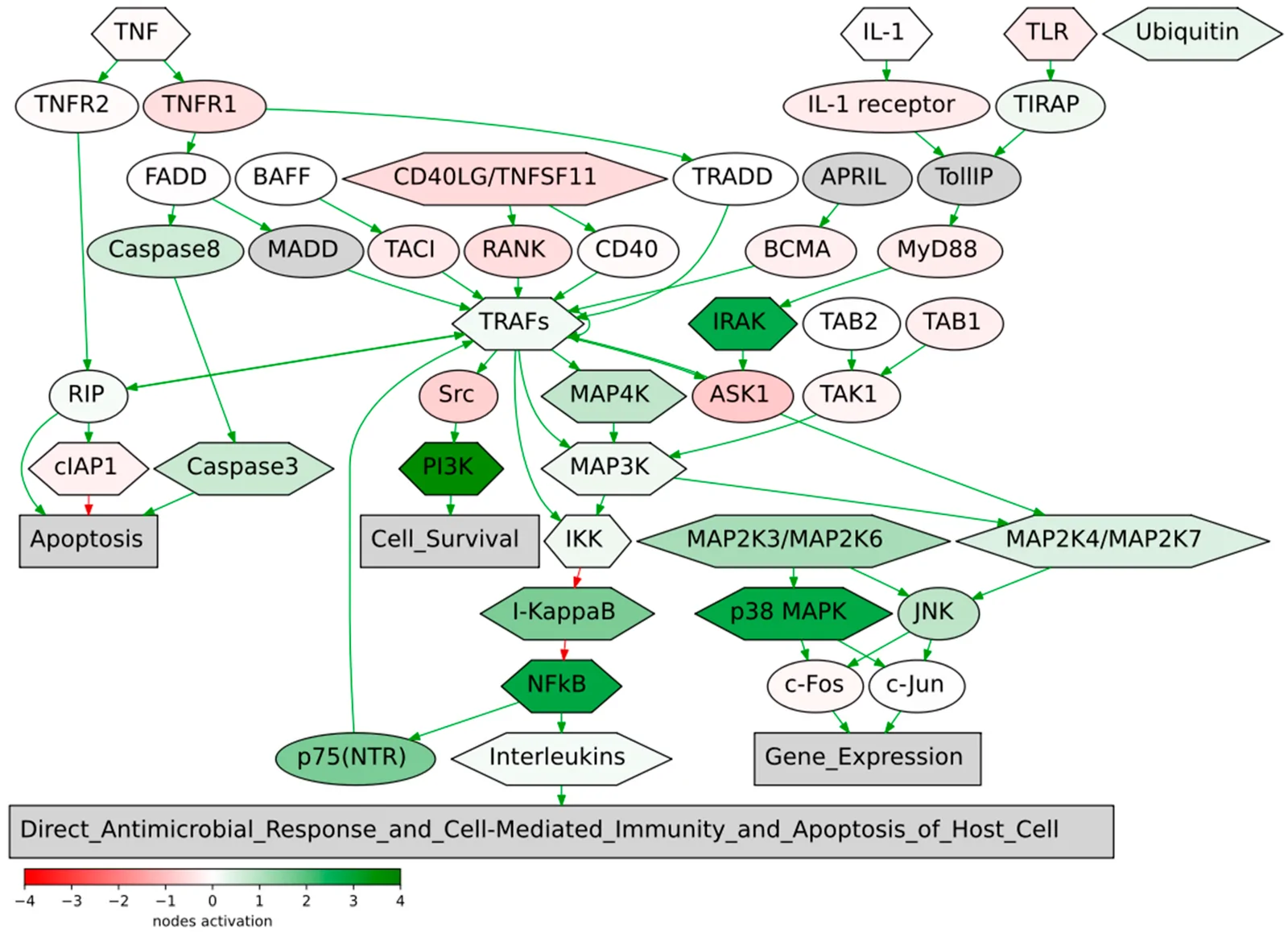
TRAF pathway activation diagram in response to Polyphemusin III. Green and red arrows correspond to node activation and inhibition, respectively. The color intensity of each node in the graph corresponds to the logarithm of the experimental-to-control ratio of gene expression.

**Figure 6:**
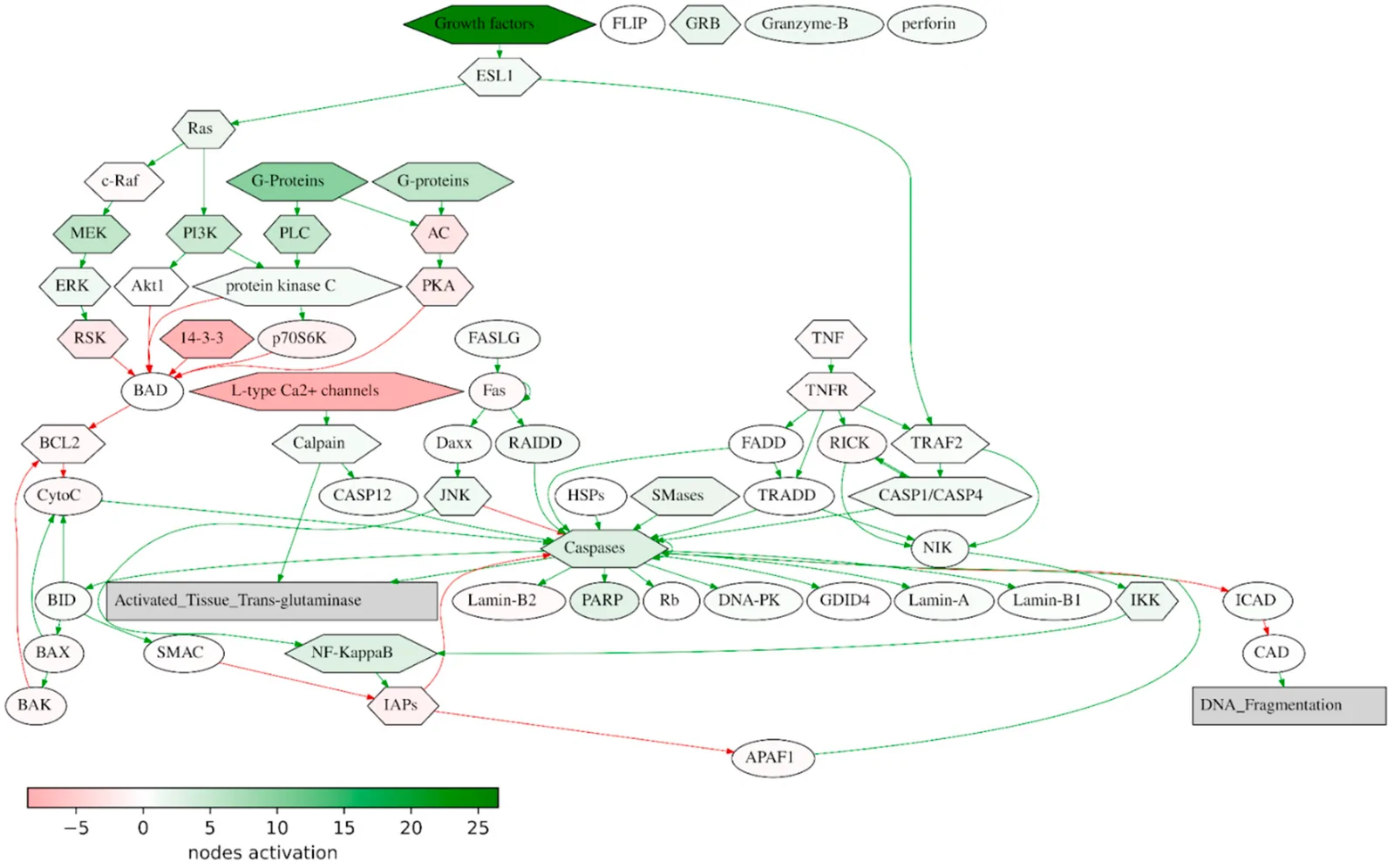
Caspase cascade pathway activation diagram in response to Polyphemusin III. Green and red arrows correspond to node activation and inhibition, respectively. The color intensity of each node in the graph corresponds to the logarithm of the experimental-to-control ratio of gene expression.

Previously, we showed that Polyphemusin III exhibits cytotoxic activity by causing Z-VAD-FMK-independent necrotic-like death in HL-60 cells [26]. According to the pathway activation profiling data, Polyphemusin III showed the most significant effect on signaling pathways associated with necrosis and necroptosis, including tumor necrosis factor receptor-associated factors (PAL = 8.4) (Figure 5), while the Caspase cascade pathway associated with apoptosis was instead suppressed with PAL = −20.36 (Figure 6).

**Figure 7:**
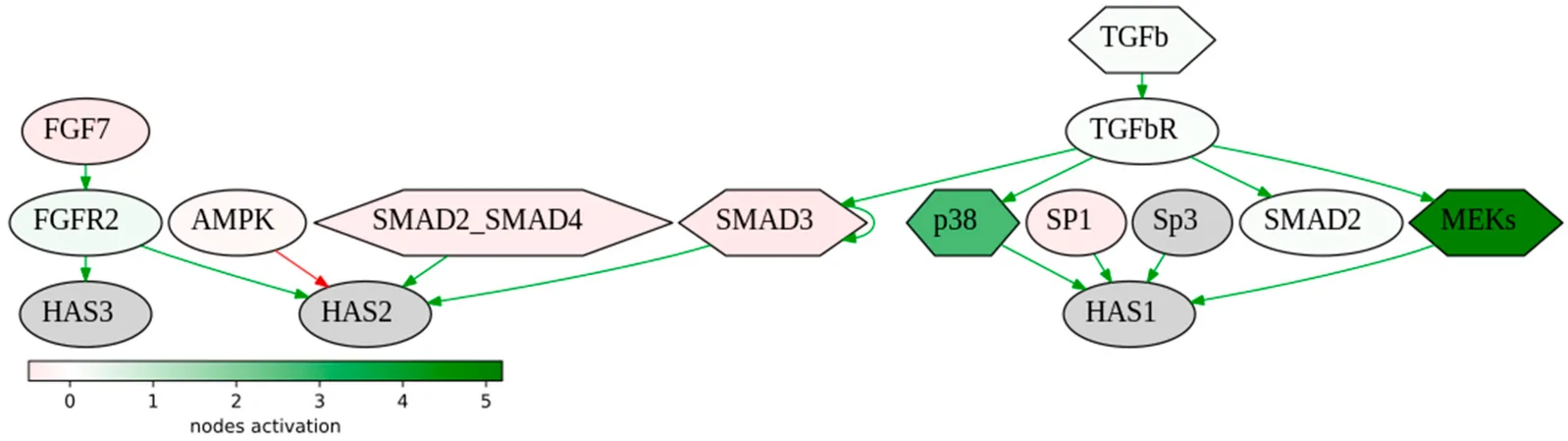
Hyaluronic acid pathway activation diagram in response to Polyphemusin III. Green and red arrows correspond to node activation and inhibition, respectively. The color intensity of each node in the graph corresponds to the logarithm of the experimental-to-control ratio of gene expression.

Furthermore, we also detected activation of the Hyaluronic acid synthesis pathway (PAL = 21.7, Figure 7), which can be explained by the protective role of this molecule in preventing necrotic cell death [35].

In summary, the obtained pathway activation data align with the known mechanisms of the cytotoxic effect of cryptophycins [28] and polyphemusines [26] studied in vitro. This indicates that the Oncobox pathway analysis platform may be a viable method for decoding the mechanisms of activity of biomolecules and biomass extracts.

### 2.4. Detailed Analysis of Activity of Extracts from Desmidiales Strains

**Figure 8:**
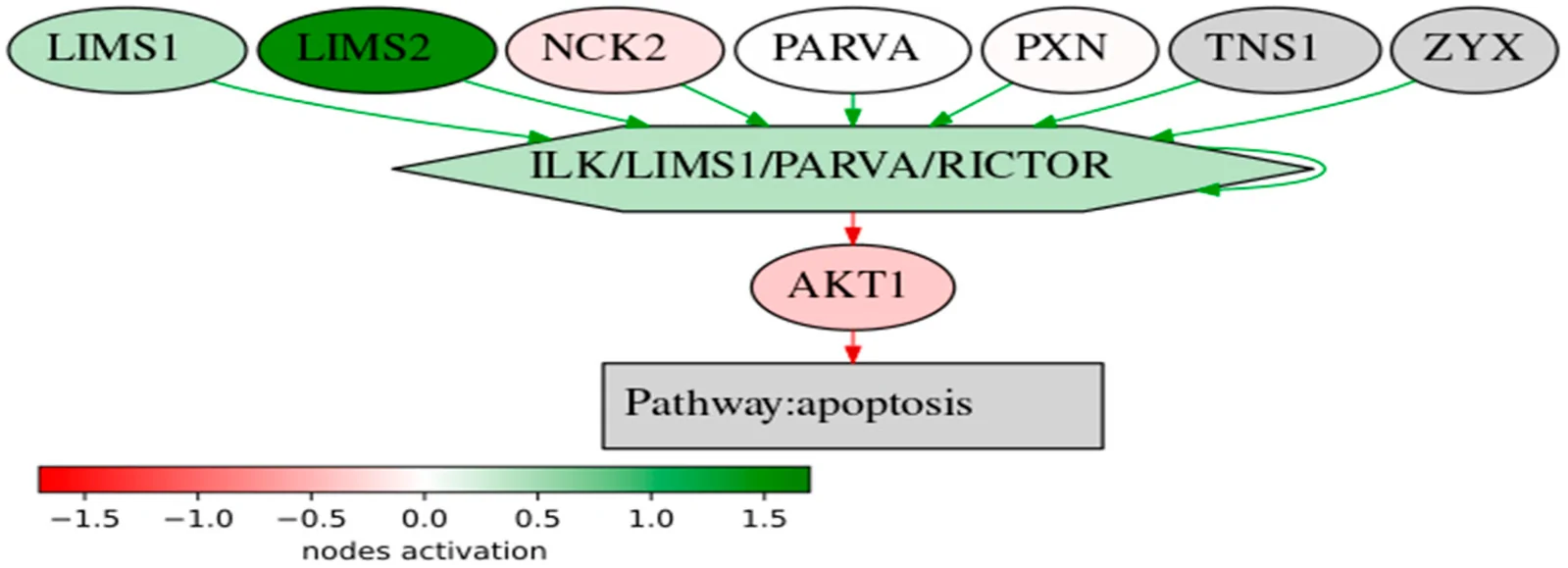
Chemokine (gene expression and apoptosis via ELK1) pathway activation diagram in response to the extract from *Cosmarium* sp. SBV90. Green and red arrows correspond to node activation and inhibition, respectively. The color intensity of each node in the graph corresponds to the logarithm of the experimental-to-control ratio of gene expression.

**Figure 9:**
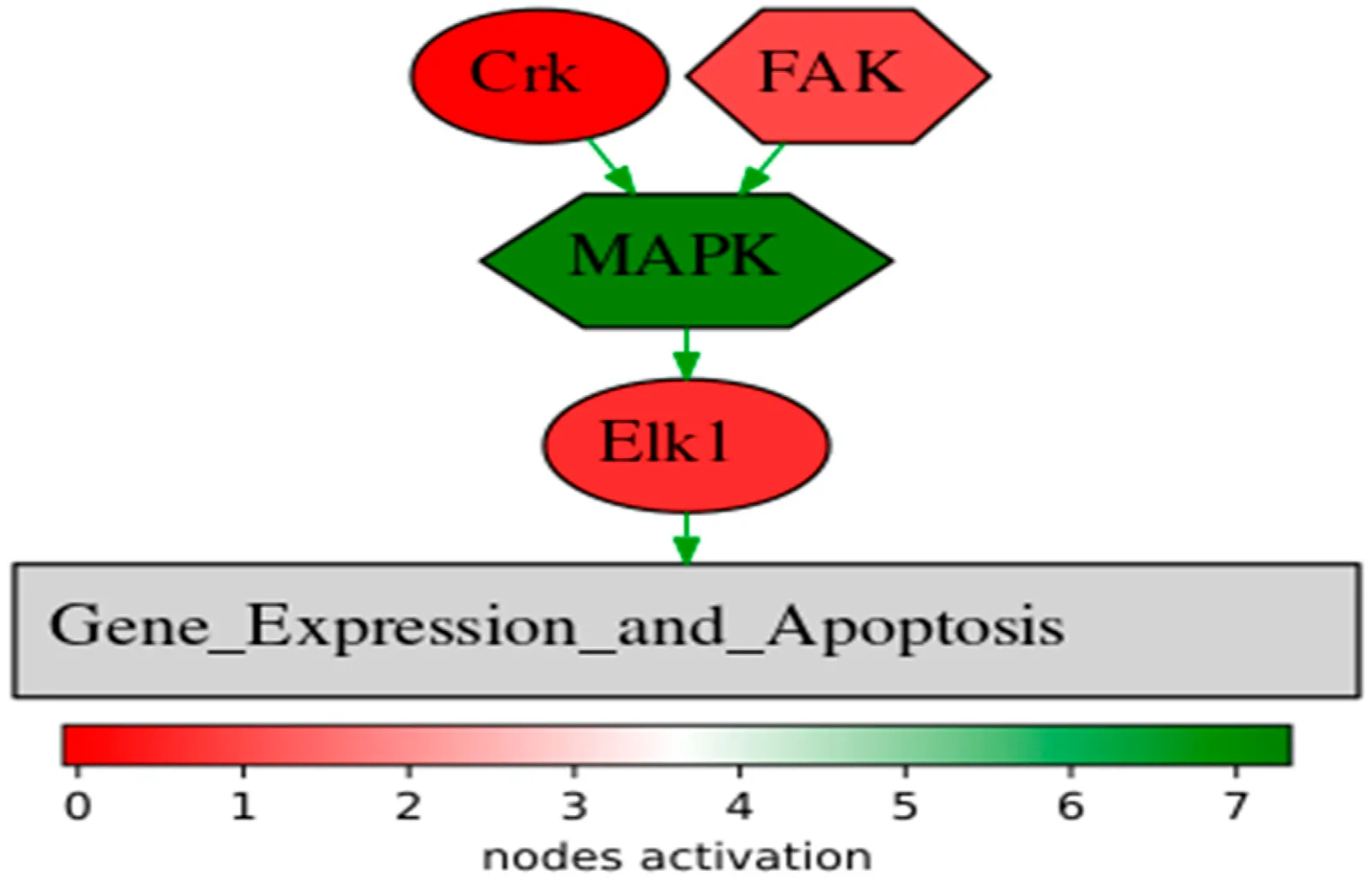
NCI Integrin-linked kinase pathway activation diagram in response to the extract from *Closterium* sp. SBV86. Green arrows correspond to node activation and inhibition, respectively. The color intensity of each node in the graph corresponds to the logarithm of the experimental-to-control ratio of gene expression.

The cytotoxic extracts of *Spondylosium planum* SBV92, *Closterium* sp. SBV86, and *Cosmarium* sp. SBV90 were of our particular interest. These strains are closely related and belong to the Order Desmidiales. Prior to the present study, no antitumor activity had been reported in these microalgae. Extracts from these strains exhibited moderate cytotoxicity in the MTT assay, with IC_50_ values of 388 ± 9 μg/mL (*Cosmarium* sp. SBV90)*,* 461 ± 31.3 μg/mL (*Closterium* sp. SBV86)*,* and 807 ± 7 μg/mL (*Spondylosium planum* SBV92). According to an Oncobox analysis, cells incubated with *Closterium* sp. SBV86 and *Cosmarium* sp. SBV90 extracts exhibited activation of at least one pathway associated with cell death, while incubation with *Spondylosium planum* SBV92 extract did not lead to activation of such pathways. In particular, *Cosmarium* sp. SBV90 extract activated the Chemokine pathway of gene expression and apoptosis via ELK1 (PAL = 39.36) (Figure 8), while *Closterium* sp. SBV86 extract activated NCI Integrin-linked kinase pathway (PAL = 24.31) in affected HL-60 cells (Figure 9).

**Figure 10:**
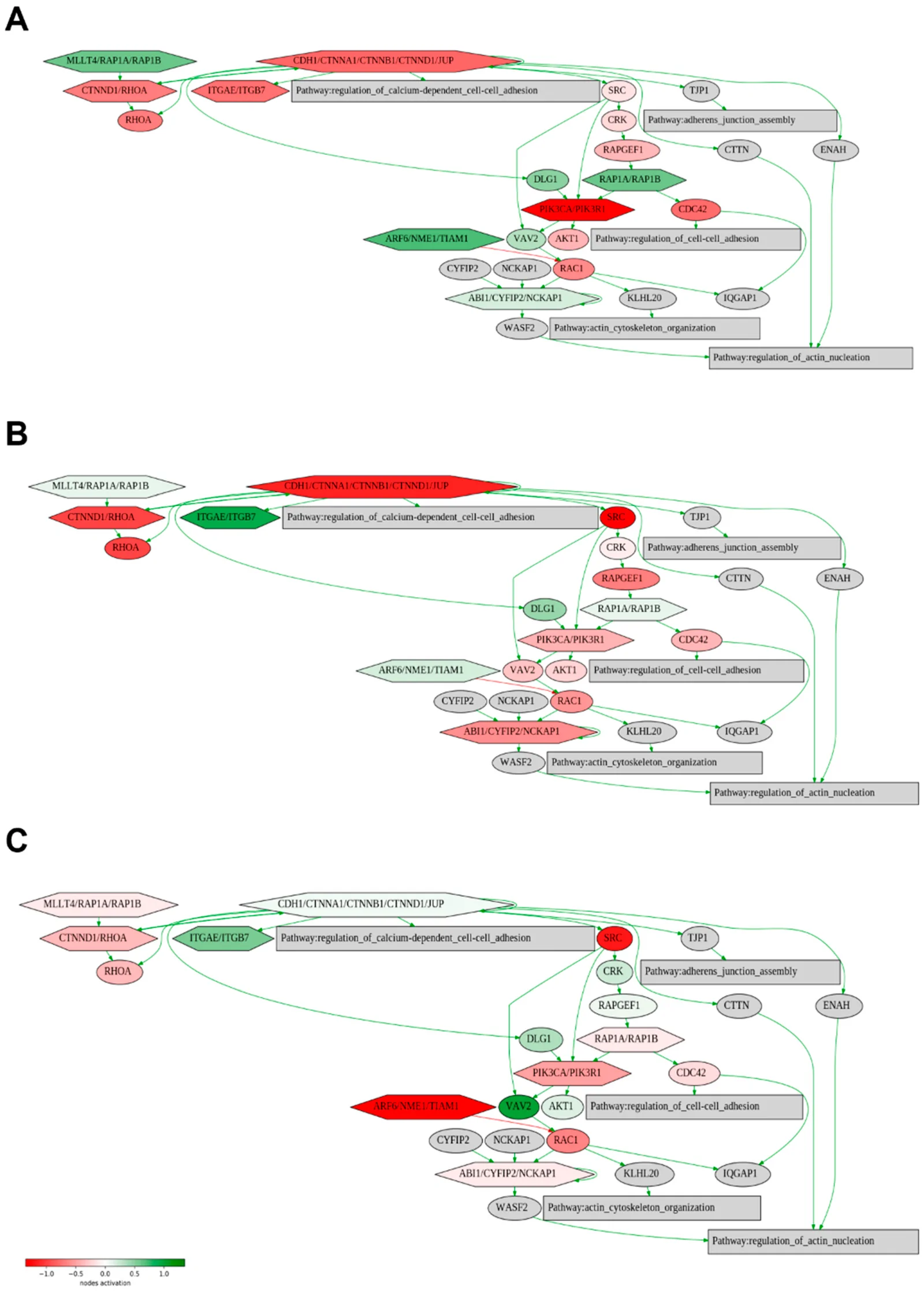
NCI E cadherin signaling in the nascent adherents junction main pathway activation diagram in response to the extract from *Cosmarium* sp. SBV90 (**A**), *Closterium* sp. SBV86 (**B**), and *Spondylosium planum* SBV92 (**C**). Green and red arrows correspond to node activation and inhibition, respectively. The color intensity of each node in the graph corresponds to the logarithm of the experimental-to-control ratio of gene expression.

Analysis of the complete pathway activation data revealed that all three extracts caused inhibition of NCI E cadherin signaling in the nascent adherents junction pathway in HL-60 cells compared to the control samples: with PAL values of −6.68 (*Cosmarium* sp. SBV90), −13.49 (*Closterium* sp. SBV86), and −0.93 (*Spondylosium planum* SBV92), as shown in Figure 10.

While NCI E cadherin signaling in the nascent adherents junction main pathway is not directly associated with cell death, it plays an important role in regulation of various cell processes, especially cell adhesion and mobility. The pathway activation profile shows that extracts from *Cosmarium* sp. SBV90, *Closterium* sp. SBV86, and *Spondylosium planum* SBV92 inhibit several oncogenes and proto-oncogenes, such as RHOA, CDC42, SRC, PIK3CA, and RAC1. Mutations and overexpression of these genes are known to promote uncontrollable cell division, metastasis, and drug resistance in various cancer types [36,37,38,39,40], thus making them viable targets for anticancer therapy.

## 3. Discussion

To extensively validate the biotechnological potential of the Oncobox platform for the purpose of studying photosynthetic microorganisms, we selected 28 strains of algae from various classes and 20 strains of cyanobacteria from our culture collection. These strains were chosen to encompass a wide range of phylogenetic and biochemical diversity. Class Chlorophyceae is very species-diverse and is frequently used in biotechnological research [41,42]. From this group, we selected ten strains from the genera *Chlamydomonas*, *Bracteacoccus*, *Scenedesmus*, *Acutodesmus*, *Haematococcus*, and *Chlorococcum* to represent the diversity within the class. Trebouxiophyceae representatives are also frequently used in biotechnology [43,44,45,46], so we selected species from the genera *Coccomyxa*, *Parietochloris*, *Picochlorum*, *Stichococcus*, *Chlorella*, *Nephrochlamys*, and *Franceia*. Despite being one of the most diverse classes of green algae, with over 4000 species, Zygnematophyceae has received comparatively limited attention in the context of biotechnological research. To close this gap, we selected several genera that differ in morphology and phylogenetic position: *Gonatozygon*, *Closterium*, *Penium*, *Spondylosium*, and *Cosmarium*. Among Bacillariophyceae, Chrysophyceae, Eustigmatophyceae, and Rhodophyceae, we selected model strains that had already demonstrated high yields of important compounds such as polyunsaturated fatty acids (PUFAs) and fucoxanthin. Among Cyanoprokaryota, we selected genera from different phylogenetic lines to cover the wide diversity of this group: *Nostoc*, *Pseudanabaena*, *Cylindrospermopsis*, *Arthrospira*, and *Oscillatoria*.

Cyanobacteria have been recognized as a potent source of bioactive metabolites of various classes, including glycolipids, macrolides, peptides, polysaccharides, and polyketides. A considerable number of these compounds have exhibited anticancer properties, including cytotoxicity, antineoplastic, and antiproliferative activity, suggesting potential pharmacological applications [47,48,49].

In the present study, two out of twenty cyanobacterial extracts demonstrated cytotoxic activity: *Pseudanabaena mucicola* SBV55, and *Nostoc* sp. SBV48. The antitumor activity of strains belonging to the genus *Pseudanabaena* has been documented in several scientific publications; this activity has been attributed to peptides in the case of *Pseudanabaena galeata* CCNP1313 [50,51]. In the present study, the cytotoxicity (IC_50_) of extracts from those cyanobacteria was 828 μg/mL and was found to be significantly lower than that of the control compound Polyfemusin III (IC_50_ = 2.5 μg/mL).

Conversely, the extract of *Nostoc* sp. SBV48 exhibited remarkably elevated activity (IC_50_ = 0.3 ng/mL), which is several orders of magnitude higher than that of the Polyfemusin III and antitumor agents commonly used in clinical practice [52]. The active metabolite of this extract was identified and purified; its chemical structure was determined to be that of the cyclic depsipeptide Cryptophycin-1. Cryptophycins, toxins characteristic of the *Nostoc* genus, demonstrate fungicidal and antitumor activity. The exposure of eukaryotic cells to low concentrations of cryptophycins results in a strong suppression of the microtubule dynamics, which leads to rapid apoptosis without a prolonged mitotic arrest. As a result of microtubule homeostasis disturbance, the apoptosis effector enzyme caspase 3 and the phosphorylation of Bcl2 (an apoptosis suppressor) are activated [52]. Bcl2 phosphorylation leads to its suppression, which has been observed in our study in Mitochondrial apoptotic pathway analysis (Figure 3). Aftermath of microtubule homeostasis disturbance was also evident by activation of Rearrangement of the cytoskeleton pathway (Figure 4). Cryptophycin-52 was investigated as an antitumor agent LY355703 in phase 1 and 2 clinical trials. Due to its narrow therapeutic window, the compound was discontinued from phase 2 clinical trials [30,52]. In recent efforts to address this limitation, researchers have explored the use of conjugates with antibodies for targeted delivery of cryptophycins to the tumor [30,52].

Among the eukaryotic microalgae examined in this study, extracts from the biomass of microalgae belonging to the genera *Phaeodactylum*, *Mallomonas*, *Stichococcus, Closterium*, *Cosmarium*, and *Spondylosium* exhibited antitumor activity. *Phaeodactylum tricornutum* is a microalga that has been utilized in the biotechnology industry for the production of omega-3 PUFAs and the carotenoid fucoxanthin. The extant literature demonstrates the antitumor activity of *P. tricornutum* extracts; however, there is no consensus on its mechanism. Researchers attribute this activity to various intracellular metabolites, including polysaccharides [53,54], alkenes [55], and the strain’s main commercial bioproduct fucoxanthin [56]. *Mallomonas furtiva* SBV-13, similar to *P. tricornutum*, has been established as a highly effective producer of fucoxanthin. The strain is distinguished by an exceptionally high content of this metabolite in biomass, which is 2.6 times higher than in *P. tricornutum* [21]. Our study was the first to demonstrate antitumor activity for strains of the genus *Mallomonas*. As with *Phaeodactylum*, the antitumor activity of *M. furtiva* SBV-13 can be attributed to fucoxanthin, for which there is substantial evidence of anticancer activity across various tumor cell lines [56,57,58]. The antitumor activity of strains belonging to the genus *Stichococcus* has been attributed to the lipid fraction in the case of *Stichococcus bacillaris* UTEX2542 [55].

Desmid algae have received little attention from a biotechnological perspective. Due to their slow growth rate in laboratory conditions, research on them has largely been descriptive, although some strains have recently been considered models for studying the sexual reproduction of microalgae [59,60]. Our research was the first to demonstrate the antitumor activity of these algae (Table 2), with all desmid strains included in our panel exhibiting it. Extracts of *Closterium* sp. SBV86 and *Cosmarium* sp. SBV90 induced apoptosis pathways in the test cells (see Figure 8 and Figure 9), while the extract of *Spondylosium planum* SBV92 did not demonstrate such activity. Concurrently, all three extracts exhibited a similar profile of activity on the NCI E cadherin signaling pathway in the nascent adherents junction main pathway, which is associated with cell adhesion and migration (Figure 10). A subsequent analysis of the nodes revealed the capacity of extracts from these strains to inhibit the expression of several important oncogenes and proto-oncogenes within this pathway. This finding has the potential to serve as a foundation for further research on desmid microalgae as a promising source of antitumor compounds.

Microarray-based profiling of gene expression was selected because the present study required quantitative measurement of a predefined gene set for molecular pathway reconstruction rather than discovery of novel transcripts. Customized in-house microarrays enabled standardized and cost-effective parallel profiling of the relatively large experimental panel while providing the expression data required for Oncobox pathway analysis. This strategy is conceptually consistent with large-scale perturbational transcriptomic approaches, such as the Connectivity Map, where microarray-based gene expression profiling was successfully used to characterize cellular responses to large panels of bioactive compounds [61].

Based on the obtained results, it can be concluded that the Oncobox pathway analysis platform enables highly reliable prediction of biological activity of microalgae metabolites that affect human cell viability and cause changes in transcriptional regulation. Two approaches can be implemented to analyze the obtained data. First, the greater the number of affected molecular pathways, the more pronounced the cytotoxic activity of the compound under study will be (an extract from *Nostoc* sp. SBV48 is an example of this concept). Conversely, if the extract under study affects only a few molecular pathways, this may indicate high specificity to certain intracellular targets (an extracts from *Closterium* sp. SBV86 and *Spondylosium planum* SBV92 are examples of this concept).

Thus, the main methodological advance of this study is the integration of pathway-level transcriptomic profiling with phenotypic screening, enabling both prioritization of active natural extracts and generation of hypotheses regarding their mechanisms of action within the same experimental workflow.

A limitation of the present study is that functional screening was performed using a single tumor cell line, HL-60; therefore, the observed activities should be interpreted as HL-60-specific until validated in additional tumor models and normal human cells. The presented method’s applications extend beyond the search for antitumor compounds. Experimentally established differential activation of intracellular signaling pathways can be compared with existing datasets of intracellular pathway profiles to predict other types of biological activity. CLUE (Connectopedia: The CLUE Knowledge Base, URL: https://clue.io/connectopedia/, accessed on 20 May 2026) is an example of such a database. It contains pathway activation data obtained in response to the action of thousands of drugs on various cell lines, which could be used to search for natural compounds with similar activities.

However, interpretation of such similarity analyses for crude microalgal extracts requires caution because each extract may contain multiple biologically active components. The resulting transcriptional and pathway activation profiles may therefore represent combined, potentially additive, synergistic, or antagonistic effects of several compounds and may not correspond to the profile of any single reference compound. Thus, clustering or similarity analysis against reference perturbational databases should primarily be considered a hypothesis-generating approach to prioritizing candidate mechanisms and compounds for subsequent investigation.

Moreover, optimization of the composition of profiled genes used for sample analysis can increase the accuracy of selecting strains with different biological activities. Thus, using omics technologies to quantitatively assess the level of activation of intracellular molecular pathways allows for the accelerated isolation of new biologically active substances from microalgae biomass.

## 4. Methods

### 4.1. Microalgae and Cyanobacteria Cultivation and Extract Preparation

Forty-eight species of microalgae and cyanobacteria from our culture collection were used in the present study. Molecular identification of strains was performed using 18S and 16S rRNA sequencing as described in our previous studies [21,43]. Microalgae and cyanobacteria were cultivated for 14 days in a Multitron laboratory incubator-shaker (Infors HT, Bottmingen, Switzerland) at 24 °C with constant shaking at 150 rpm and 5% CO_2_ in air supply. Light intensity was set to 160 μmol/m^2^/s, and the photoperiod was set to 16 h of light and 8 h of darkness. Cultures were algologically pure, containing no other algae, microalgae, cyanobacteria or fungi cells.

Freshwater microalgae strains were cultivated in a modified WC medium [62] with a ten-fold content of nitrate and phosphate. The medium contained 0.85 g/L NaNO_3_; 0.114 g/L K_2_HPO_4_ ∙ 3H_2_O; 0.0126 g/L NaHCO_3_; 0.0368 g/L CaCl_2_ ∙ 2H_2_O; 0.0212 g/L Na_2_SiO_3_ ∙ 9H_2_O; 0.5 g/L Tris; 0.037 g/L MgSO_4_ ∙ 7H_2_O; microelements (4.36 mg/L Na_2_EDTA; 3.15 mg/L FeCl_3_ ∙ 6H_2_O; 0.01 mg/L CuSO_4_ ∙ 5H_2_O; 0.022 mg/L ZnSO_4_ ∙ 7H_2_O; 0.01 mg/L CoCl_2_ ∙ 6H_2_O; 0.18 mg/L MnCl_2_ ∙ 4H2O; 0.006 mg/L Na_2_MoO_4_ ∙ 2H_2_O; 1 mg/L H_3_BO_3_) and vitamins (0.1 mg/L thiamin, 0.0005 mg/L biotin).

Freshwater cyanobacteria strains were cultivated in a BG-11 medium [63]. The medium contained 1.5 g/L NaNO_3_; 0.04 g/L K_2_HPO_4_ ∙ 3H_2_O; 0.075 g/L MgSO_4_ ∙ 7H_2_O; 0.036 g/L CaCl_2_ ∙ 2H_2_O; 0.006 g/L citric acid, 0.006 g/L ammonium ferric citrate; 0.001 mg/L Na_2_EDTA-Mg; 0.02 g/L Na_2_CO_3_; microelements (0.28 mg/L H_3_BO_3_; 0.18 mg/L MnCl_2_ ∙ 4H_2_O; 0.022 mg/L ZnSO_4_ ∙ 7H_2_O; 0.004 mg/L Na_2_MoO_4_ ∙ 2H_2_O; 0.008 mg/L CuSO_4_ ∙ 5H_2_O; 0.005 mg/L Co(NO_3_)_2_ ∙ 6H_2_O) and vitamins (0.1 mg/L thiamin, 0.0005 mg/L biotin).

Saltwater microalgae and cyanobacteria strains were cultivated in a modified F/2 medium [64,65] with a ten-fold content of nitrate and phosphate. The medium contained 0.75 g/L NaNO_3_; 0.05 g/L NaH_2_PO_4_ ∙ H_2_O; 0.03 g/L Na_2_SiO_3_ ∙ 9H_2_O; 0.0044 g/L Na_2_EDTA; 0.0031 g/L FeCl_3_ ∙ 6H_2_O; 10.37 g/L NaCl; 1.72 Na_2_SO_4_; 0.29 g/L KCl; 0.075 g/L NaHCO_3_; 0.037 g/L NaBr; 0.011 g/L H_3_BO_3_; 0.0013 g/L NaF; 4.69 g/L MgCl_2_ ∙ 6H_2_O; 0.658 g/L CaCl_2_ ∙ 2H_2_O; 0.01 g/L SrCl_2_ ∙ 6H_2_O, microelements (0.008 mg/L CuSO_4_ ∙ 5H_2_O; 0.022 mg/L ZnSO_4_ ∙ 7H_2_O; 0.02 mg/L CoCl_2_ ∙ 6H_2_O; 0.18 mg/L MnCl_2_ ∙ 4H_2_O; 0.004 mg/L Na_2_MoO_4_ ∙ 2H_2_O) and vitamins (0.1 mg/L thiamin, 0.0005 mg/L biotin).

After 14 days, the biomass was separated by centrifugation, then lyophilized for 24 h in a FreeZone lyophilizer (Labconco, Kansas City, MO, USA) and stored at −70 °C. An ethanol extract with concentration was prepared from the lyophilizate using a ratio of five parts alcohol to one part biomass (which equaled to a stock concentration of 200 mg/mL). After adding the ethanol, the sample was homogenized with glass beads and left in the dark for one hour. After one hour, the cellular debris and beads were separated by centrifugation. Following the centrifugation process, the supernatant was subjected to a two-stage filtration procedure using Spin-X (Corning, Corning, NY, USA) 2 mL centrifuge tubes with a 0.22 micron filter membrane. The resulting filtrate was then transported on the same day for experimentation on cells.

### 4.2. Human Cell Culturing

The human leukemia cell line HL-60 was obtained from the American Type Culture Collection (ATCC, Manassas, VA, USA). HL-60 cells were cultured in RPMI-1640, supplemented with 10% heat-inactivated fetal bovine serum, 2 mM *L*-glutamine, 100 μg/mL penicillin, and 100 U/mL of streptomycin (all—Gibco, New York, NY, USA) at 37 °C in a 5% CO_2_ atmosphere. HL-60 cells were maintained at low passage numbers and routinely checked for mycoplasma by PCR.

### 4.3. Functional Testing of Microalgae and Cyanobacteria Extracts

HL-60 cells were incubated in 96-well round-bottom tissue culture plates (2 × 10^4^ cells/well, Greiner, Kremsmünster, Austria) with 48 microalgae and cyanobacteria extracts for 6 h under standard culture conditions. Stock solutions of extracts with a concentration of 200 mg/mL were dissolved in the medium and added to the plates using serial dilutions. The final concentrations of extracts in the wells were 1000, 500, 250, 125, 63, 32, 16, and 8 μg/mL. A medium without extracts was used as a negative control. A medium containing Polyphemusin III at a concentration of 50 mM/mL was used as a positive control [63].

After incubation, total RNAs were isolated from HL-60 cell samples to screen for gene expression profiles. Cells incubated with the antimicrobial peptide Polyphemusin III were used as a positive control of cytotoxicity [63] and untreated cells were used as the norms. One million of HL-60 cells were used for each sample preparation, and total RNA preps were isolated using the RNeasy Mini Kit (Qiagen, Hilden, Germany). RNA quality control was performed using an Agilent 2100 Bioanalyzer (Santa Clara, CA, USA). RNA concentration was measured using the RNA 6000 Nano or Qubit RNA Assay Kit (Invitrogen, Carlsbad, CA, USA). Three independent experiments were performed for each extract.

### 4.4. Gene Expression Profiling and Analysis

Gene expression was profiled according to [66] using microarray hybridization of the RNA samples obtained. The expression levels of 2211 genes were measured in each sample. The data underwent quantile normalization using the R library “preprocessCore”.

B3 synthesizer (CustomArray, Redmond, WA, USA) was utilized to synthesize oligonucleotide probes on Custom Array ECD 4X2K/12K chips. The Complete Whole Transcriptome Amplification WTA2 Kit (Sigma, Burlington, MA, USA) was utilized for the reverse transcription and amplification of libraries. The manufacturer’s protocol was modified by adding a mixture of biotinylated dUTP to the dNTP amplification reaction, resulting in a final dTTP/biotin-dUTP ratio of 5/1. Hybridization was performed according to the CustomArray ElectraSenseTM protocol. The hybridization mixture contained 2.5 μg of labeled DNA library, 6X SSPE, 0.05% Tween-20, 20 mM EDTA, 5X Denhardt’s solution, 100 ng/μL sonicated calf thymus gDNA, and 0.05% SDS. The hybridization mixture was incubated with the chip overnight at 50° C. Hybridization efficiency was assessed electrochemically using the CustomArray ElectraSense Detection KitTM and the ElectraSenseTM 4X2K/12K reader.

The differential gene expression analysis was performed using the R package LIMMA (version 3.52.4) [67]. Samples treated with the extracts under analysis were compared with a control group of samples.

### 4.5. Molecular Pathway Activation Analysis

The Pathway Activation Level (PAL) values were calculated for the quantile-normalized gene expression data. PAL calculation is expressed by the formula:

$${PAL}_{p}=\sum {ARR}_{np}\times {BTIF}_{n}\times ln({CNR}_{n})$$

where

*CNR_n_* (case-to-normal ratio) is the ratio of the expression level of gene *n* in the sample under study to the geometric mean expression level of this gene in the control samples.

The Boolean flag *BTIF_n_* (beyond tolerance interval flag) is set to zero if the *CNR_n_* does not pass the significance threshold, i.e., when the difference with the control group is not significant (*p* > 0.05).

*ARRnp* (activator/repressor role of gene *n* in pathway *p*) is a discrete value equal to:

−1 when the product of gene *n* is a repressor of pathway *p*;

1 when the product of gene *n* is an activator of pathway *p*;

0 when the product of gene *n* exhibits ambivalent activity in pathway *p* or when its activity is unknown;

0.5 and −0.5, respectively, when gene product *n* is predominantly an activator or repressor of pathway *p*.

PAL calculations were performed using the OncoboxPD pathways database [62], with seven control samples used for gene expression normalization. The control samples were the expression profiles of cell lines that were not exposed to extracts. Differentially activated pathways were identified using the Mann−Whitney test implemented in the Python SciPy library (version 1.14). The results of differential activation analysis were visualized using Volcano plots with Cohen’s d as an effect size metrics. Pathway activation schemes were visualized using the OncoboxPD software (version 3.22.3) [62]. One-way analysis of variance (ANOVA) was performed to identify pathways which activation differed between subgroups of samples treated with different concentrations of the testing substance(s) using the Python SciPy library (version 1.14) [68].

### 4.6. Cell Viability Assay

Viability of HL-60 cells was analyzed by colorimetric MTT (3-[[4,5]-dimethylthiazol-2-yl]-2,5-diphenyltetrazolium bromide (Sigma-Aldrich, Burlington, MA, USA)) assay [69]. HL-60 cells were cultured in 96-well round-bottom tissue culture plates (2 × 10^4^ cells/well, Greiner, Kremsmünster, Austria) with 48 microalgae and cyanobacteria extracts for 72 h under standard culture conditions. Stock solutions of extracts with a concentration of 200 mg/mL were dissolved in the medium and added to the plates using serial dilutions. The final concentrations of extracts in the wells were 1000, 500, 250, 125, 63, 32, 16, and 8 μg/mL. A medium without extracts was used as a negative control. A medium containing Polyphemusin III at a concentration of 50 mM/mL was used as a positive control [63].

Following incubation, the MTT solution (final concentration 250 μg/mL) was added to each sample for 3 h. Reaction optical density (OD) was assessed by Multiscan FC microplate reader at a wavelength of 540 nm. Cell viability was calculated using the formula (OD*_treated cells_* − OD*_blank_*)/(OD*_control cells_* − OD*_blank_*) × 100%, where OD*_blank_* represents OD in control wells containing no cells. SigmaPlot (version 14.0, Systat Software Inc., Evanston, IL, USA) was used to generate dose–response curves. All MTT experiments were reproduced at least three times.

### 4.7. Isolation and Purification of Cytotoxic Metabolite from Nostoc *sp.* SBV48

Cyanobacterial cells were eliminated from a dark-colored suspension of the culture broth of the cyanobacteria strain by centrifugation for 20 min at 7000 rpm (Beckman J2-21 centrifuge, Brea, CA, USA) and filtration through 0.47 μm GF/A and 0.22 μm GPWP filters (Millipore, Burlington, MA, USA). Cleared supernatant was diluted by bidistilled water 1:1 and the sample volume of 1 L were loaded on a 5 g cartridge packed with Strata C18-E, 55 μm, 70 Å sorbent (Phenomenex, Torrance, CA, USA) at a flow rate of 15 mL/min using a peristaltic pump (Masterflex L/S variable speed pump Systems, Masterflex, Gelsenkirchen, Germany). Extraction by water−acetonitrile mixtures was performed at a flow rate of 15 mL/min; four fractions of 15 mL were collected sequentially, with 10%, 50%, and two factions of 100% acetonitrile. Biological activities of the fractions were tested on HEK293T and HL-60 cells. The most active fraction #3 was further analyzed by HPLC on RP column using the Nexera X2 LC 30A instrument (Shimadzu, Kyoto, Japan) equipped with SPD-M20A detector (Supplementary Figure S2). HPLC fractions were collected and tested for activity, and the fraction containing pure active substance (the peak at retention time 8 min) was isolated and then analyzed by LCMS. Through repeated HPLC isolations, approximately 1 mg of the substance of interest was obtained for NMR structure determination and subsequent biological assays.

#### 4.7.1. Lcms Analysis of Cytotoxic Metabolite from *Nostoc* sp. SBV48

LC-MS analysis was carried out on ACQUITY UPLC H-Class System (Waters Corporation, Milford, MA, USA) equipped with a ACQUITY UPLC BEH C18 Column (1.7 µm, 130Å, 50 mm × 2.1 mm), TUV, and SQD-ESI detectors. Samples were eluted with a H2O-MeCN linear gradient (from 50 to 100% of MeCN for 5 min, 450 µL/min) with 0.1% formic acid as an eluent additive. UV data were collected at 220 nm, MS scans were performed within the 50–1050 Da and 1000–2000 Da ranges in positive mode. As a result of the analysis of the pure active fraction, an ion with *m*/*z* = 655.6 Da, presumably the molecular ion [M+H]^+^, and its dimer [2M+H]^+^ with *m*/*z* = 1310.0 were registered (Supplementary Figure S3).

#### 4.7.2. Structure Elucidation of Cytotoxic Metabolite from *Nostoc* sp. SBV48

The structure of the compound was elucidated using the conventional heteronuclear NMR approach. The purified sample was dissolved in 330 uL of DMSO-d6 (Sigma Aldrich, Burlington, MA, USA) and placed into a 5 mm Shigemi tube. To elucidate the structure of the compound, the following NMR spectra were recorded: 1D ^1^H, 1D ^13^C, multiplicity-edited ^13^C-HSQC, ^15^N-HSQC, DQF-COSY, ^13^C-HSQC-TOCSY, ^13^C-HMBC (two spectra, optimized for J_CH_ equal to 5 and 8 Hz), ^15^N-HMBC (optimized for J_HN_ = 5 Hz), and ROESY (200 ms mixing time). Spectra were recorded using the Bruker Avance III 800 spectrometer, equipped with a cryogenic TCI probe (Bruker Biospin Gmbh, Ettlingen, Germany) at 30 °C. The obtained spectra were analyzed manually using the standard approach in the MestreNova-NMR software (version 16.0.0).

### 4.8. Statistical and Quantitative Analysis

MTT experiments were independently reproduced at least three times, and quantitative cytotoxicity data are presented as mean ± SD. IC_50_ values were determined from dose–response curves using SigmaPlot. The performance of pathway-based cytotoxicity prediction was evaluated by direct comparison with the MTT assay across all 48 tested extracts and was expressed as the proportion of concordant classifications.

## 5. Conclusions

We examined the effect of 48 microalgae and cyanobacteria extracts on the viability of HL-60 human lymphoma cells and identified 8 extracts that can inhibit tumor cell growth. The precision of predicting antitumor activity based on signaling pathway analysis was 92%. In order to verify the ability of Oncobox to identify the molecular mechanisms of biological activity, an in-depth analysis of pathway activation data was conducted for the most cytotoxic extract from *Nostoc* sp. SBV48 and the antitumor/antimicrobial peptide Polyphemusin III. The mechanisms of action of these compounds were successfully deciphered based on Oncobox data, which coincided with those described in the literature.

Furthermore, this study provides a novel account of the antitumor activity of three Desmid strains of genera *Closterium*, *Cosmarium*, and *Spondylosium*. In addition to cytotoxic activity, the study found that all three Desmid extracts exhibit an analogous activity profile on a number of oncogenes and proto-oncogenes belonging to the NCI E cadherin signaling in the nascent adherents junction main pathway, which is associated with cell adhesion and migration.

## Figures and Tables

**Table 1:** Classes of studied photosynthetic microorganisms.

Eukaryotic Photosynthetic Microorganisms	Number of Strains
Chlorophyceae	6
Zygnematophyceae	8
Trebouxiophyceae	8
Bacillariophyceae	3
Chrysophyceae	1
Eustigmatophyceae	1
Rhodophyceae	1
Prokaryotic Photosynthetic Microorganisms	Number of Strains
Cyanoprokaryota	20

**Table 2:** Samples exhibiting cytotoxic activity.

Strain	IC_50,_ μg/mL	Number of Activated Molecular Pathways Associated with Cell Death	Total Number of Affected Molecular Pathways
*Nostoc* sp. SBV48	0.00032 ± 0.00001	25	290
*Phaeodactylum tricornutum* Bohlin SBV20	280 ± 55.5	6	39
*Closterium* sp. SBV86	461 ± 31.3	1	25
*Spondylosium planum* (Wolle) W.West & G.S.West SBV92	807 ± 7	1	6
*Mallomonas furtiva* Gusev, Certnerová, Škaloudová & Škaloud SBV13	114 ± 5	0	60
*Cosmarium* sp. SBV90	388 ± 9	0	6
*Stichococcus* sp. SBV40	764 ± 34	0	24
*Pseudanabaena mucicola* Nauman & Huber-Pestalozzi SBV55	828 ± 10	0	9
Polyphemusin III	2.5 ± 0.1	3	103

IC_50_ values are means ± SD of three replicate groups (*n* = 3).

## Data Availability

The original contributions presented in this study are included in the article/Supplementary Material. Further inquiries can be directed to the corresponding author.

## Supplementary Materials

The following supporting information can be downloaded at: https://www.mdpi.com/article/10.3390/ph19091440/s1, Figure S1: 1D 13C NMR spectrum of T_08092017 with the signal assignment (split into fragments for readability); Figure S2: HPLC analysis of solid phase extraction fraction #3. HPLC conditions: column Agilent Zorbax SB C18 9.2 × 150 mm, 5 μm, eluent solvent A—water, solvent B—MeCN; gradient elution from 70 to 90% of solvent B; flow rate 2 mL/min; UV detection at 280 nm. Active fraction corresponds to shaded peak at tR 8 min. (UV spectrum of the peak is at the right); Figure S3: Mass-spectra of the active substance within 50–1000 and 1000–2000 ranges in positive mode; Table S1: Species of studied photosynthetic microorganisms; Table S2: NMR chemical shift assignment.
