# Supplementary material for: Integrated Cell-Based Transcriptomic and Pathway Profiling Enables Scalable Functional Screening of Bioactive Microalgal Extracts
Source: Pharmaceuticals (Basel). 2026 Sep 11;19(9):1440. doi: 10.3390/ph19091440 (PMC13610492; doi:10.3390/ph19091440)
Supplement: Supplementary file 1 [file pharmaceuticals-19-01440-s001.zip › Supplementary Table S1.pdf]

Supplementary Table S1. Species of studied photosynthetic microorganisms

Collection number	Species	Class
SBV20	*Phaeodactylum tricornutum* Bohlin	Bacillariophyceae
SBV106	*Odontella aurita* (Lyngbye) C.Agardh	Bacillariophyceae
SBV24	*Halamphora sp.*	Bacillariophyceae
SBV53	*Chlamydomonas sp.*	Chlorophyceae
SBV61	*Bracteacoccus bullatus* Fuciková, Flechtner & L.A.Lewis	Chlorophyceae
SBV68	*Chlorococcum sp.*	Chlorophyceae
SBV6	*Haematococcus pluvialis* Flotow	Chlorophyceae
SBV34	*Scenedesmus costatus* Schmidle	Chlorophyceae
SBV33	*Acutodesmus obliquus* (Turpin) Hegewald & Hanagata	Chlorophyceae
SBV13	*Mallomonas furtiva* Gusev, Certnerová, Škaloudová & Škaloud	Chrysophyceae
SBV21	*Nannochloropsis oculata* (Droop) D.J.Hibberd	Eustigmatophyceae
SBV60	*Porphyridium purpureum* (Bory) K.M.Drew & R.Ross	Rhodophyceae
SBV62	*Coccomyxa sp.*	Trebouxiophyceae
SBV69	*Parietochloris grandis* Y.Maltsev & Maltseva	Trebouxiophyceae
SBV71	*Nephrochlamys sp.*	Trebouxiophyceae
SBV1	*Chlorella vulgaris* Beijerinck	Trebouxiophyceae
SBV4	*Chlorella vulgaris* Beijerinck	Trebouxiophyceae
SBV67	*Franceia sp.*	Trebouxiophyceae
SBV95	*Picochlorum sp.*	Trebouxiophyceae
SBV40	*Stichococcus sp.*	Trebouxiophyceae
SBV92	*Spondylosium planum* (Wolle) West & G.S.West	Zygnematophyceae
SBV145	*Cosmarium sp.*	Zygnematophyceae
SBV144	*Cosmarium sp.*	Zygnematophyceae
SBV86	*Closterium sp.*	Zygnematophyceae
SBV93	*Penium margaritaceum* Brébisson ex Ralfs	Zygnematophyceae
SBV150	*Closterium sp.*	Zygnematophyceae
SBV90	*Cosmarium sp.*	Zygnematophyceae
SBV87	*Gonatozygon sp.*	Zygnematophyceae
SBV45	*Drouetiella sp.*	Cyanoprokaryota
SBV103	*Limnospira maxima* (Setchell & N.L.Gardner) Nowicka-Krawczyk, Mühlsteinová & Hauer	Cyanoprokaryota
SBV147	*Limnospira sp.*	Cyanoprokaryota
SBV102	*Cylindrospermopsis raciborskii* (Wołoszyńska) Seenayya & Subba Raju	Cyanoprokaryota
SBV36	*Oscillatoria sp.*	Cyanoprokaryota
SBV119	*Limnothrix sp.*	Cyanoprokaryota
SBV149	*Nostoc sp.*	Cyanoprokaryota
SBV146	*Nostoc sp.*	Cyanoprokaryota

SBV48	*Nostoc sp.*	Cyanoprokaryota
SBV139	*Oscillatoria sp.*	Cyanoprokaryota
SBV158	*Oscillatoria sp.*	Cyanoprokaryota
SBV141	*Oscillatoria sp.*	Cyanoprokaryota
SBV58	*Oscillatoria sp.*	Cyanoprokaryota
SBV157	*Oscillatoria sp.*	Cyanoprokaryota
SBV43	*Pseudanabaena mucicola* Nauman & Huber-Pestalozzi	Cyanoprokaryota
SBV55	*Pseudanabaena mucicola* Nauman & Huber-Pestalozzi	Cyanoprokaryota
SBV44	*Pseudanabaena tremula* J.R.Johansen & Casamatta	Cyanoprokaryota
SBV56	*Pseudanabaena sp.*	Cyanoprokaryota
SBV42	*Pseudanabaena sp.*	Cyanoprokaryota
