# Supplementary material for: Integrated Cell-Based Transcriptomic and Pathway Profiling Enables Scalable Functional Screening of Bioactive Microalgal Extracts
Source: Pharmaceuticals (Basel). 2026 Sep 11;19(9):1440. doi: 10.3390/ph19091440 (PMC13610492; doi:10.3390/ph19091440)
Supplement: Supplementary file 1 [file pharmaceuticals-19-01440-s001.zip › Supplementary Table S2.pdf]

Supplementary Table S2. NMR chemical shift assignment**.**

Atom	$^{13}C/^{15}N$	^{1}H	HMBC[a]	COSY[b]
2	170.47			
3	70.95	4.90(dd[c])	1',2,2',5	1'
5	175.74			
6	37.73	2.73(m)	6a,5,7,8	6a,7
6a	14.64	1.01(d)	5,6,7	6
7	39.65	3.43;3.16	5,6,6a,8,9	8,6
8	102.54	7.23 (dd)	6,7,9,10	7
9	171.41			
10	56.01	4.28(ddd)	7",1",11,8,9,12	7",11
11	121.4	8.36(d, 8Hz)	12,10,7"	10
12	165.47			
13	125.68	5.81 (d,15Hz)	14,15,12	14
14	140.55	6.46(ddd)	13,12,15,16	13,15
15	37.09	2.72;2.30	16,1'",14,13	14,16
16	76.41	5.14(ddd)	14,15,1'",2'",2	15,1'"
1'	39.37	1.55;1.29	2,3,2',3',4'	3,2'
2'	24.27	1.62(m)	1',3',4',3	3',4'.1'
3'	22.94	0.79(d)	1',2',4'	2'
4'	21.63	0.89(d)	1',2',3'	2'
№	13C/15N	1H	HMBC[a]	COSY[b]
1"	131.95			
2"	130.67	7.31(d, 2Hz)	6",4", 7"	6"
3"	120.95			
4"	153.55			
4"a	56.42	3.83(s)	4"	
5"	113.19	7.06(d, 8.5Hz)	1",3"	6"
6"	129.01	7.19 (dd)	4",2",7"	5"
7"	35.23	3.03;2.69	9,10,11,1",2",6"	

1'''	40.22	1.83(m)	15,16,1'''a,2''',3'''	16,1'''a,2'''
1'''a	14.06	1.06(d)	16,1''',2'''	1'''
2'''	63.17	3.00(dd)	1''',16,1'''a,3''',4'''	1''',3'''
3'''	58.50	3.91 (d,3.2Hz)	4''',5/9''',2''',1'''	2'''
4'''	137.71			
5/9'''	126.35	7.33(d,8Hz)	3''',7''',5/9'''	
6/8'''	129.12	7.39(dd)	4''',8'''	
7'''	128.79	7.36 (dd)	5/9'''	

[a]The correlation between the current proton and nitrogen/carbon nuclei in the other positions in HMBC spectra are shown.

[b]Interproton cross-peaks in DQF-COSY

[c]The signal multiplicity and J-couplings are shown in parentheses. s- singled, d - doubleton, dd - doubleton of doubletons, m - multiplet more complicated than ddd.
